# Shift work and use of psychotropic medicine: a follow-up study with register linkage

**DOI:** 10.5271/sjweh.3872

**Published:** 2020-07-01

**Authors:** Karen Albertsen, Harald Hannerz, Fil Lic, Martin L Nielsen, Anne Helene Garde

**Affiliations:** TeamArbejdsliv ApS, Valby, Denmark; National Research Centre for the Working Environment, Copenhagen, Denmark; Lægekonsulenten.dk, AS3 Companies, Viby J, Denmark; Department of Public Health, University of Copenhagen, Copenhagen K, Denmark

**Keywords:** Key terms antidepressant, anxiolytic, mental health, prescription drug, occupational health, sedative, shift worker

## Abstract

**Objective:**

This study aimed to investigate a prospective association between shift work and use of psychotropic medicine.

**Methods:**

Survey data from random samples of the general working population of Denmark (N=19 259) were linked to data from national registers. Poisson regression was used for analyses of prospective associations between shift work and redeemed prescriptions of psychotropic medicine. Prevalent cases were excluded at baseline. In secondary analyses, we tested differential effects on subsets of psychotropic medicine and, cross-sectionally, we studied correspondence between estimates based on psychotropic medicine and self-reported mental health. According to the protocol we interpret results from the secondary analyses following the principles for nested hypothesis testing, if the primary analyses reject the null-hypothesis, and otherwise we regard it as hypothesis generating exploratory analyses.

**Results:**

In the primary analysis, the rate ratio for incidence of psychotropic medicine among shift workers was 1.09 (95% confidence interval 0.99–1.21). Results from the secondary analyses suggested increased incidence of use of hypnotics, sedatives and antidepressants and decreased incidence of use of anxiolytics. Cross-sectional analysis suggested increased risk for use of psychotropic medicine (all kinds), but not for poor self-rated mental health.

**Conclusions:**

Results did not support that working in shifts to the extent that is currently practiced in Denmark is associated with an increased incidence of overall psychotropic medicine use. Future studies should test, whether there is a differential incidence for different drugs among shift workers as suggested by the secondary analyses and how psychotropic medicine use and mental health are related.

Shift work is related to disturbances in the circadian rhythm and sleep disturbances, which are suspected to contribute to mental health problems (1–4). A meta-analysis of 11 primary studies concluded that night shift work was significantly associated with an increased risk of depression (5), and a prospective study found a ‘flexible/non-regulated’ schedule prospectively associated with antidepressant prescription among females (6). Other prospective studies have not found shift work associated with the development of mental health problems (7, 8) nor with use of psychotropic drugs (9–11). Thus, it is essential to investigate the possible negative effects of shift work on mental health.

There may be different mechanisms behind a possible association between shift work and mental health. Circadian misalignment may disrupt the internal synchronization of the hypothalamic-pituitary-adrenal (HPA) axis, that is responsible for several neurotransmitters and hormones (1). In turn this may lead to abnormal responses to stress. And together with sleep disturbances, the stress responses may affect mood, and vigilance (12), and as such the regulation of emotions (1). Shiftwork is furthermore known to have negative impact on work-life balance, social life issues (13, 14) and on marital satisfaction (15). And beside this, some of the occupational sectors where shift work is prevalent, eg, healthcare and protective services, are also sectors chracterized by increased risk for exposure to traumatic events (16, 17). Thus, biological as well as social and environmental factors may interact as underlying mechanisms behind an association between shift work and mental health.

The aim of the present study was to assess if shift work is associated with increased risk of psychotropic medicine use. Firstly, we investigated the prospective association between shift work and incident use of psychotropic medicine. Secondly, we tested differential prospective effects on subsets of psychotropic medicine. Thirdly, in order to evaluate whether prescription bias was present, we related the association between shift work and self-rated mental ill health and medicine use, respectively.

## Methods

The data material, the hypotheses and the statistical methods of the study were defined, peer-reviewed and published in a detailed study protocol (18), before we performed the linkage between the exposure and the outcome data of the study. For information about the data material and statistical methods and models see Hannerz & Albertsen (18, 19). Only a brief description of the data and methods will be given here.

### Data material

The data material was obtained through a linkage of survey data from the Copenhagen Psychosocial Questionnaire study (COPSOQ) sample of 2004, the Danish National Working Environment Survey (DANES) of 2008, and the Danish Work Environment Cohort Study (DWECS) of 1995, 2000, 2005, and 2010 with data from the Central Person Register, the Employment Classification Module, and the Danish National Prescription Registry (DNPR). The COPSOQ study sample is a random sample, which comprises 4732 people, 20–59 years of age, whereof 3517 are wage earners (20). DANES is based on a random sample of the Danish population in 2008. It comprises responses from 6531 persons 18–59 years of age, of which 4919 are employees. The DWECS is an open cohort study, based on a random sample of people 18–59 year of age in the Danish population. The cohort contains a representative cross-sectional data-collection among at least 5000 employees every fifth year from 1990–2010 (21).

### Primary analysis

*Case definition*. A person was defined as a case if and when he or she redeemed a prescription for drugs in the anatomical therapeutic chemical (ATC) code category N05 [psycholeptica=antipsychotics (N=58), anxiolytics (N=495), hypnotics and sedatives (N=752)] or N06 [psychoanaleptica=antidepressants (N=941), psychostimulants (N=12) and anti-dementia drugs (N=0)]. In addition, 656 cases received combinations of different drugs. In total there were 2914 cases. For specification of this see (19).

*Follow-up and inclusion criteria*. Each of the included samples was followed for a period of 2–5 years (depending on the time available between the sampling and the end of the study period, 31 December 2012) beginning at the start of the calendar year succeeding the one in which they were sampled. Participants who redeemed a prescription for a medication with an ATC-code that belong to the case definition, during the calendar year preceding baseline (prevalent cases) were excluded from the follow-up. Participants could participate in several rounds. A participant who reached the clinical endpoint of the study was not allowed to re-enter the follow-up, ie, there would be maximum one case per person. People aged 21–59 years at the start of the follow-up period and employed ≥32 weekly working hours around the time of the interview were included [for further information see (18)].

*Exposure assessment*. The surveys contain information on the participants’ normal work schedules. The questions and response categories vary slightly between the questionnaires, but all of them can identify workers who are either on fixed night shifts or rotational shift work schedules (see supplementary material, www.sjweh.fi/show_abstract.php?abstract_id=3872, appendix 1 and 2).

*Statistical model*. Poisson regression was used to model incidence rates of redeemed prescriptions for psychotropic medicine as a function of currently working in shifts (permanent night shifts, rotational shift work schedules (both with and without night shifts) or irregularly placed hours versus permanent day, morning or evening). The analysis was adjusted for sex, age (10-year classes), sample (DWECS 1995; DWECS 2000; COPSOQ 2004; DWECS 2005; DANES 2008; DWECS 2010), weekly working hours (32–40, 41–48, >48 hours/week) and socioeconomic status. The logarithm of person years at risk was used as offset. The significance level was set to 0.05. A likelihood ratio test was used to test the null hypothesis.

### Secondary analyses

As described in the protocol (18), we performed a series of secondary analyses (for results from all analyses see appendix 3). Sensitivity analyses were performed in the same way as we did in the primary analysis, but with endpoints defined by the subsets of N05B (anxiolytics), N05C (hypnotics and sedatives), and N06A (antidepressants). In order to evaluate whether prescription bias was present, we compared the association between shift work and self-rated mental ill health (MHI-5; cut-off: 52 points) with the association between shift work and redeemed prescriptions. Logistic regression was used to model the odds of outcomes in cross-sectional analyses. The analyses were adjusted like in the primary analysis. In the protocol (18) it was described that interpretation of results from the secondary analyses would follow the principles for nested hypothesis testing, if the primary analyses rejected the null-hypothesis and otherwise, they would be regarded as hypothesis generating exploratory analyses.

## Results

In the primary analysis, the inclusion criteria for age, employment status and working hours were fulfilled for 29 837 observations. Of these, we excluded 3084 due to prior redeemed case prescriptions, and 794 due to missing data on shift work, which left us with 25 959 observations (19 259 persons) to be included in the analysis. The included observations yielded a total of 2914 new cases of psychotropic drug use in 99 019 person years at risk. For details of each of the included data sets see (19).

In the prospective analysis, we found no overall increased risk for incidence of psychotropic drug use among shift workers compared to fixed day workers (P=0.09). Thus, the likelihood ratio test did not reject the null hypothesis. The estimated rate ratio, person years at risk and number of cases are given in table 1.

**Table 1:** The estimated prospective rate ratio (RR) for incident use of any type psychotropic drugs [CI=confidence interval; Pyrs=person years of risk].

Population	Shift work	Pyrs	Cases	RR ^a^	95% CI
All employees	Yes	13 812	440	1.09	0.99–1.21
	No	85 207	2474	1.00	

a The analysis was controlled for sex, age, socioeconomic status, working hours, and sample.

In the sensitivity analysis (see table 2), we observed higher incidence for hypnotics and sedatives and antidepressants, and lower incidence for anxiolytics.

**Table 2:** Rate ratio (RR) with 95% confidence interval (CI) for incident use of anxiolytics, hypnotics and sedatives, and antidepressants, as a function of shift work (yes vs no) among employees in Denmark 1996–2012.

Endpoint	Shift work	Person years	Cases	RR ^a^	95% CI
N05B anxiolytics	Yes	15 653	141	0.86	0.72–1.02
	No	95 039	973	1.00	
N05C hypnotics and sedatives	Yes	15 481	230	1.21	1.05–1.40
	No	94 348	1188	1.00	
N06A antidepressants	Yes	15 132	281	1.23	1.08–1.40
	No	93 308	1415	1.00	

a Adjusted for sex, age, socioeconomic status, weekly working hours and sample.

The cross-sectional analysis showed that working in shifts was associated with an increased propensity to redeem prescriptions for psychotropic drugs, but not with an increased tendency to report poor mental health (see table 3).

**Table 3:** The estimated odds ratios (OR) of the outcomes as a function of shift versus non-shift work. [CI=confidence interval]

Case definition	Shift work	Observations	Cases	OR ^a^	95% CI
Use of psychotropic medication (P=0.0058)	Yes	2561	217	1.26	1.08–1.48
	No	16 172	1178	1.00	
Poor self-rated mental health (P=0.6849)	Yes	2507	109	1.03	0.87–1.22
	No	15 842	679	1.00	

a The analyses were controlled for sex, age, socioeconomic status, working hours, and sample.

## Discussion

In the primary analysis, we did not find a statistically significant association between shift work and the incidence of psychotropic drug usage (all types combined) among Danish employees. Given that this study had enough power to detect an effect, the primary results support a conclusion saying that working in shifts to the extent that is currently practiced in Denmark is not associated with an increased incidence of overall psychotropic medicine use. The overall result is in line with findings from other prospective studies that did not find work in shift associated with development of mental health problems (7, 8) or with use of psychotropics (9–11). As mentioned in the introduction, some previous prospective studies have found associations between shift work and psychotropic drug usage. However, one of these studies (6) was based on analyses with rather low statistical power and multiple tests, of which only one reached significance. The other (5) only included two prospective studies in a meta-analysis – one of which was severely underpowered and the other reported in an untransparent way without inclusion of the prospective findings. The authors recommend studies with stronger designs in order to draw confirmative relationships.

It is important to notice that results from this study do not address whether working in shifts for some people may be contraindicated due to the experience of mental health problems (eg, serious sleeping problems). In line with the findings of this study, results from a large non-randomized pseudo-trial from Finland (22) showed no increased risk of developing common mental disorder after changing from non-night to night work. The authors found, however, (i) increased likelihood of recovery from common mental disorder if night workers changed from night- to non-night work and that (ii) night workers were more likely to change to non-night work if they had developed common mental disorder. Thus, the selection out of night work may be dependent on the individuals’ experience of mental health problems (including sleeping disorders). Thus, employees whose mental well-being is affected are likely to change their schedules to day work, and this change is – in turn – likely to help them recover from the common mental disorder.

Results from the secondary analyses suggested increased incidence for the use of hypnotics, sedatives, and antidepressants and decreased incidence for the use of anxiolytics among shift workers. A simple interpretation of the differential effects may be that it was a coincidence. Another interpretation could be that shift work increases the incidence of sleeping problems and depression and decreases the incidence of anxiety. These oppositely directed effects cancel out each other and give a non-significant total effect. If this interpretation is right, different incidences of diagnoses among shift workers versus others should be hypothesized. Both interpretations should be tested in future studies by including both several psychotropic drugs and diagnoses as outcomes. A third interpretation could be that there are different practices for prescription of psychotropics to people working in shifts compared to non-shift workers. The general practitioner may decide the specific medication for mental health and sleeping problems among people working in shifts taking into consideration the requirement of often being awake and not being too tired at night and being able to sleep during the day. As one of the side-effects of anxiolytics may be drowsiness, it is likely that the general practitioner will rather prescribe antidepressants over anxiolytics to people working in shifts and suffering from anxiety. This interpretation will get support if studies find no difference in diagnoses of depression and anxiety among shift workers versus non-shift workers. Results from a survey on the use of psychotropic medication in the general populations of France, Germany, Italy, and the United Kingdom lend support to this interpretation: “Subjects said that they were taking an antidepressant to reduce depression in 30.7% of cases; 25.5% said it was to help them to sleep; in 24.5% it was to reduce anxiety; and in 13.4% it was to help them to sleep and to reduce anxiety and depression” (23). Thus, the prescription of psychotropics is not always specific for specific sufferings.

Cross-sectional results suggested increased prevalence of psychotropic drug use but not poor mental health among shift workers. These results may reflect that the medication has had a positive effect on the perception of mental health. Therefore, we cannot be sure whether the increased prevalence of drug use was due to an increased need of treatment for mental health problems or an increased propensity to seek treatment (eg, for sleeping problems). We can only conclude that the two outcomes give different estimates of risk. As suggested above, future studies may also shed more light on possible prescription bias. Clarifying the relation between shift work and changes in mental health and drug use would probably require frequent follow-up on both outcomes or more clinical studies.

### Strengths and weaknesses

Within-study selection bias was eliminated through our study protocol, in which hypotheses and statistical models were specified, peer reviewed, and published before the questionnaire data were linked to the registers. The study population was randomly sampled from the target population and the statistical power was sufficiently large to detect important effects. The problem with reversed causality was minimized through the prospective design and the exclusion of prevalent cases. Bias from incomplete follow-up data was eliminated by use of a clinical endpoint that was ascertained through national registers, which cover all residents of the target population. As suggested by results from previous research, selection processes into (24) as well as out of shift work (22) are likely, and perception of mental health and sleep quality may play a role in both selection processes. A limitation of the study was that it included neither measures of the length of exposure or previous daytime work among the shift workers nor previous shift work among the non-shift workers. Due to this limitation, the results cannot rule out a potential dose–response effect of shift work on mental health. For further methodological considerations see (19).

### Concluding remarks

Results did not support that working in shifts – to the extent that is currently practiced in Denmark – is associated with an increased incidence of overall psychotropic medicine use. Future studies should test whether there is a differential incidence for different drugs among shift workers as suggested by the secondary analyses and if there is prescription bias for the outcome of psychotropic medicine.

## Supplementary material

Supplementary material
