# Supplementary material for: Shift work and use of psychotropic medicine: a follow-up study with register linkage
Source: Scand J Work Environ Health. 2020 Jul 1;46(4):350–5. doi: 10.5271/sjweh.3872 (PMC8506318; doi:10.5271/sjweh.3872)
Supplement: Supplementary material [file SJWEH-46-350-S001.pdf]

APPENDIX 1: THE WORDING (IN DANISH) OF THE QUESTIONS USED TO OBTAIN INFORMATION ON WORKING HOURS AND WORK SCHEDULES

Weekly working hours:

DWECS, 1995

(De næste spørgsmål handler om Deres nuværende arbejde. Hvis De har flere jobs, vil vi gerne spørge om Deres hovedbeskæftigelse som lønmodtager (den de bruger flest timer på). Hvis De ikke er i arbejde i øjeblikket, beder vi Dem svare vedrørende Deres seneste job.)

Hvor mange timer om ugen arbejder De normalt i Deres hovedjob som lønmodtager? Antal timer om ugen:______

Og i Deres bijob? Antal timer om ugen:______

(Hovedjobbet er det job. IP bruger flest timer på uanset om det er 10 eller 37 timer. Hvis flere bijob, tælles alle timerne i bijob sammen. Hvis timeantallet varierer, må det spørges om et omtrent gennemsnit over en måned.)

DWECS, 2000

(De næste spørgsmål handler om dit nuværende arbejde som lønmodtager. Hvis du har flere jobs, vil vi gerne spørge om din hovedbeskæftigelse som lønmodtager (den du bruger flest timer på). Hvis du ikke er i arbejde i øjeblikket, beskriv dit seneste job.)

Hvor mange timer om ugen arbejder du normalt i dit hovedjob, inkl. eventuelle fastlagte timer, betalt overarbejde og andet ekstraarbejde, fx hjemmearbejde? Angiv timer i gennemsnit: _ _ _

Hvor mange timer om ugen arbejder du normalt i dit bijob, inkl. eventuelle fastlagte timer, betalt overarbejde og andet ekstraarbejde, fx hjemmearbejde? Angiv timer i gennemsnit: _ _ _

DWECS, 2005

(De næste spørgsmål handler om dit nuværende arbejde som lønmodtager. Hvis du har flere jobs, vil vi gerne at du beskriver din hovedbeskæftigelse som lønmodtager (den du bruger flest timer på). Hvis du ikke er i arbejde i øjeblikket, beskriv dit seneste job.)

Hvor mange timer om ugen arbejder du normalt i din hovedbeskæftigelse, inkl. eventuelle fastlagte timer, betalt overarbejde og andet ekstraarbejde, fx hjemmearbejde? I gennemsnit _ _ _ timer _ _ minutter

Hvor mange timer om ugen arbejder du normalt i din bibeskæftigelse, inkl. eventuelle fastlagte timer, betalt overarbejde og andet ekstraarbejde, fx hjemmearbejde? I gennemsnit _ _ _ timer

DWECS, 2010
Hvor mange timer om ugen arbejder du i din hovedbeskæftigelse, inkl. eventuelle ekstratimer? Antal hele timer _ _ _

Hvor mange timer om ugen arbejder du normalt i din bibeskæftigelse? Antal hele timer _ _ _

COPSOQ, 2004
Hvor mange timer arbejder du rent faktisk om ugen, når du tæller overarbejde og bijob med? (Gennemsnit per uge i det seneste år) _ _ timer per uge

DANES, 2008
Hvor mange timer arbejder du rent faktisk om ugen i din hovedbeskæftigelse? (Skriv venligst gennemsnit per uge i det sidste år) _ _ _ timer per uge

Hvis du har et bijob, hvor mange timer arbejder du så rent faktisk om ugen i dit bijob? (Skriv venligst gennemsnit per uge i det sidste år) _ _ _ timer per uge

Work schedules:

DWECS, 1995
Hvordan er Deres arbejdstid normalt placeret? (Hovedbeskæftigelsen som lønmodtager)
Fast dagtid; Toholdsskift; Treholdsskift; Uregelmæssig placering i løbet af døgnet/ugen efter særligt arbejdsskema eller turnusordning; Fast aftenskift/aftenarbejde; Fast natskift/natarbejde; Fast morgenarbejde; Andet.

DWECS, 2000
Hvordan er din arbejdstid normalt placeret? (gælder hovedbeskæftigelsen)
Fast daghold; Toholdsskift; Treholdsskift; Uregelmæssig placering i løbet af døgnet/ugen efter særligt arbejdsskema eller turnusordning; Fast aftenskift/aftenarbejde; Fast natskift/natarbejde; Fast morgenarbejde; Andet.

DWECS, 2005

Hvordan er din arbejdstid normalt placeret? (gælder hovedbeskæftigelsen)
Dagtid eller fast daghold; Toholdsskift; Treholdsskift; Uregelmæssig placering i løbet af døgnet/ugen efter særligt arbejdsskema eller turnusordning; Fast aftenskift/aftenarbejde; Fast natskift/natarbejde; Fast morgenarbejde; Andet.

DWECS, 2010
På hvilket tidspunkt af døgnet arbejder du sædvanligvis i din hovedbeskæftigelse?
Fast dagarbejde; Fast aftenarbejde (overvejende mellem kl. 15 og 24); Fast natarbejde (overvejende mellem kl. 24 og 05); Skiftende arbejdstider med natarbejde; Skiftende arbejdstider uden natarbejde; Andet.

COPSOQ, 2004
På hvilket tidspunkt af døgnet arbejder du sædvanligvis?
Fast dagarbejde (overvejende mellem kl. 06 og 18); Fast aftenarbejde (overvejende mellem kl. 15 og 24); Fast natarbejde (overvejende mellem kl. 22 og 06); Skiftende arbejdstider uden natarbejde; Skiftende arbejdstider med natarbejde; Andet.

DANES, 2008
På hvilket tidspunkt af døgnet arbejder du sædvanligvis i din hovedbeskæftigelse?
Fast dagarbejde (overvejende mellem kl. 06 og 18); Fast aftenarbejde (overvejende mellem kl. 15 og 24); Fast natarbejde (overvejende mellem kl. 22 og 06); Skiftende arbejdstider; Andet.

APPENDIX 2: THE WORDING (TRANSLATED FROM DANISH) OF THE QUESTIONS USED TO OBTAIN INFORMATION ON WORKING HOURS AND WORK SCHEDULES

Weekly working hours:

DWECS, 1995

(The next questions are about your present work. If you have more than one job, then we only ask about your primary job as a wage earner (the job in which you work most hours). If you are not working at present, we kindly ask you to answer on basis of you last job.)

How many hours per week do you work in your primary job as a wage earner?
Number of hours per week: _________

How many hours per week do you work on the sideline?
Number of hours per week: __________

(The primary job is the job the IP spent most hours doing no matter if it is 10 or 37 hours. If he/she has several extra jobs, then the number of hours in these jobs must be added. If the number of hours varies, ask about an approximate monthly average.)

DWECS, 2000

(The next questions are about your present work. If you have more than one job, then we only ask about your primary job as a wage earner (the job in which you work most hours). If you are not working at present, we kindly ask you to answer on basis of you last job.)

How many hours per week do you work in your primary job as a wage earner?
(including scheduled hours, paid over-time, and other extra work, e.g. home work)
Average number of hours: ______

How many hours per week do you normally work on the sideline, including scheduled hours, paid overtime, and other extra work, e.g. homework? Average number of hours: ______

DWECS, 2005

(The next questions are about your current work as a wage earner. If you have multiple jobs, we would like you to describe your primary job as a wage earner (the one you use most hours on). If you are not working at the moment, describe your most recent job.)

How many hours a week do you normally work in your primary job, including any established working hours, paid overtime, and other extra work such as work from home?

On average: ______ hours ______ minutes

How many hours per week do you normally work in your second job including any established working hours, paid overtime, and other extra work such as work from home?

On average: ______ hours

DWECS, 2010

How many hours per week do you work in your primary job, including overtime?

Number of hours _______

How many hours per week do you normally work in your second job?

Number of hours _______

COPSOQ, 2004

How many hours per week do you actually work including overtime hours and other jobs?

(Please write the average per week during the last year). ____ hours per week

DANES, 2008

How many hours do you actually work per week in your primary job, including overtime?

(Please write the average per week within the last year) ____ hours per week

If you have a second job, then how many hours per week do you work in your second job? (Please write the average per week within the last year) ____ hours per week

Work schedules:

DWECS, 1995

How are your working hours normally placed?

(Your primary job as a wage earner)

Fixed day duty

Working on two shifts

Working on three shifts

Irregularly placed during the day/week according to special schedule or rotation

Fixed evening shift/evening work

Fixed night shift/night work

Fixed morning duty

Other, please write here

DWECS, 2000

How are your working hours normally placed?

(Your primary job as a wage earner)

Fixed day duty

Working on two shifts

Working on three shifts

Irregularly placed during the day/week according to special schedule or rotation

Fixed evening shift/evening work

Fixed night shift/night work

Fixed morning duty

Other, please write here

DWECS, 2005

How are your working hours normally placed?

(Your primary job)

Fixed day duty

Working on two shifts

Working on three shifts

Irregularly placed during the day/week according to special schedule or rotation

Fixed evening shift/evening work

Fixed night shift/night work

Fixed morning duty

Other

If other, indicate what:

DWECS, 2010

At what time of the day do you usually work in your primary job?

Fixed day duty

Fixed evening work (primarily between 3 pm and midnight)

Fixed night work (primarily between midnight and 5 am)

Variable working hours with night work

Variable working hours without night work

Other, write:

COPSOQ, 2004

At what time of the day do you usually work?

Fixed day duty (primarily between 6 am and 6 pm)

Fixed evening work (primarily between 3 pm and midnight)

Fixed night work (primarily between 10 pm and 6 am)

Variable working hours without night work

Variable working hours with night work

Other, write:

DANES, 2008

At what time of the day do you usually work in your primary job?

Fixed day duty (primarily between 6 am and 6 pm)

Fixed evening work (primarily between 3 pm and midnight)

Fixed night work (primarily between 10 pm and 6 am)

Variable working hours

Other, write:

Supplementary appendix 3 to: Albertsen K, Hannerz H, Nielsen ML, Garde AH. Shift work and use of psychotropic medicine: a follow-up study with register linkage

Results from exploratory subset analyses

In our study protocol, we defined a series of secondary analyses of prospective associations between shift work and use of psychotropic medication [Hannerz and Albertsen, 2014]. The results of these analyses are given in the below tables.

Table S1. Rate ratio with 95% confidence interval (CI) for incident use of psychotropic drugs as a function of shift work (Yes vs No) among employees in Denmark 1996–2012, stratified by sex, age, socioeconomic status and weekly working hours

Sub-population	Shift work	Person years	Cases	Rate ratio*	95% CI
Male employees	Yes	7359	182	1.00	0.85 - 1.17
	No	47 010	1115	1.00	-
Female employees	Yes	6453	258	1.17	1.03 - 1.34
	No	38 197	1359	1.00	-
Employees < 40 years	Yes	7035	178	0.98	0.83 - 1.15
	No	38 197	973	1.00	-
Employees >= 40 years	Yes	6777	262	1.18	1.04 - 1.35
	No	47 010	1501	1.00	-
Legislators, senior officials and managers	Yes	217	5	1.01	0.39 - 2.57
	No	3058	62	1.00	-
Professionals	Yes	1539	58	1.45	1.10 - 1.92
	No	15 111	381	1.00	-
Technicians and associate professionals	Yes	3209	107	1.20	0.97 - 1.49
	No	18 416	504	1.00	-
Employees in occupations that require skills at a basic level	Yes	6283	178	0.95	0.81 - 1.11
	No	34 147	1066	1.00	-
Employees in elementary occupations	Yes	970	48	1.55	1.12 - 2.13
	No	5766	187	1.00	-
Employees with an unknown occupation	Yes	1594	44	0.89	0.65 - 1.23
	No	8709	274	1.00	-
Employees who work > 48 hours a week	Yes	1911	72	1.35	1.04 - 1.77
	No	8546	233	1.00	-
Employees who work 41 - 48 hours a week	Yes	1962	60	1.10	0.84 - 1.45
	No	15 702	440	1.00	-
Employees who work 32 - 40 hours a week	Yes	9938	308	1.05	0.93 - 1.19
	No	60 958	1801	1.00	-

*Adjusted for (or stratified by) sex, age, socioeconomic status, weekly working hours and sample.

Reference

Hannerz H, Albertsen K. Long working hours and subsequent use of psychotropic medicine: a study protocol. JMIR Res Protoc. 2014 Sep 19;3(3):e51. doi: 10.2196/resprot.3301.
